# COVID-19–Associated Hospitalizations Among Adults During SARS-CoV-2 Delta and Omicron Variant Predominance, by Race/Ethnicity and Vaccination Status — COVID-NET, 14 States, July 2021–January 2022

**DOI:** 10.15585/mmwr.mm7112e2

**Published:** 2022-03-25

**Authors:** Christopher A. Taylor, Michael Whitaker, Onika Anglin, Jennifer Milucky, Kadam Patel, Huong Pham, Shua J. Chai, Nisha B. Alden, Kimberly Yousey-Hindes, Evan J. Anderson, Kenzie Teno, Libby Reeg, Kathryn Como-Sabetti, Molly Bleecker, Grant Barney, Nancy M. Bennett, Laurie M. Billing, Melissa Sutton, H. Keipp Talbot, Keegan McCaffrey, Fiona P. Havers, COVID-NET Surveillance Team, COVID-NET Surveillance Team, Gretchen Rothrock, Millen Tsegaye, Julie Plano, Kyle Openo, Andy Weigel, Chloe Brown, Erica Bye, Wickliffe Omondi, Alison Muse, Christina Felsen, Eli Shiltz, Nasreen Abdullah, William Schaffner, Melanie Crossland

**Affiliations:** ^1^CDC COVID-19 Emergency Response Team; ^2^General Dynamics Information Technology, Atlanta, Georgia; ^3^California Emerging Infections Program, Oakland, California; ^4^Career Epidemiology Field Officer Program, CDC; ^5^Colorado Department of Public Health & Environment; ^6^Connecticut Emerging Infections Program, Yale School of Public Health, New Haven, Connecticut; ^7^Emory University School of Medicine, Atlanta, Georgia; ^8^Georgia Emerging Infections Program, Georgia Department of Public Health; ^9^Atlanta Veterans Affairs Medical Center, Atlanta, Georgia; ^10^Iowa Department of Public Health; ^11^Michigan Department of Health and Human Services; ^12^Minnesota Department of Health; ^13^New Mexico Emerging Infections Program, University of New Mexico, Albuquerque, New Mexico; ^14^New York State Department of Health; ^15^University of Rochester School of Medicine and Dentistry, Rochester, New York; ^16^Ohio Department of Health; ^17^Public Health Division, Oregon Health Authority; ^18^Vanderbilt University Medical Center, Nashville, Tennessee; ^19^Utah Department of Health.; California Emerging Infections Program; Colorado Department of Public Health and Environment; Connecticut Emerging Infections Program, Yale School of Public Health; Georgia Emerging Infections Program, Georgia Department of Public Health; Division of Infectious Diseases, School of Medicine, Emory University; Iowa Department of Health; Michigan Department of Health and Human Services; Minnesota Department of Health; New Mexico Emerging Infections Program, University of New Mexico; New York State Department of Health; University of Rochester School of Medicine and Dentistry; Ohio Department of Health; Public Health Division, Oregon Health Authority; Vanderbilt University Medical Center; Salt Lake County Health Department

Beginning the week of December 19–25, 2021, the B.1.1.529 (Omicron) variant of SARS-CoV-2 (the virus that causes COVID-19) became the predominant circulating variant in the United States (i.e., accounted for >50% of sequenced isolates).* Information on the impact that booster or additional doses of COVID-19 vaccines have on preventing hospitalizations during Omicron predominance is limited. Data from the COVID-19–Associated Hospitalization Surveillance Network (COVID-NET)^†^ were analyzed to compare COVID-19–associated hospitalization rates among adults aged ≥18 years during B.1.617.2 (Delta; July 1–December 18, 2021) and Omicron (December 19, 2021–January 31, 2022) variant predominance, overall and by race/ethnicity and vaccination status. During the Omicron-predominant period, weekly COVID-19–associated hospitalization rates (hospitalizations per 100,000 adults) peaked at 38.4, compared with 15.5 during Delta predominance. Hospitalizations rates increased among all adults irrespective of vaccination status (unvaccinated, primary series only, or primary series plus a booster or additional dose). Hospitalization rates during peak Omicron circulation (January 2022) among unvaccinated adults remained 12 times the rates among vaccinated adults who received booster or additional doses and four times the rates among adults who received a primary series, but no booster or additional dose. The rate among adults who received a primary series, but no booster or additional dose, was three times the rate among adults who received a booster or additional dose. During the Omicron-predominant period, peak hospitalization rates among non-Hispanic Black (Black) adults were nearly four times the rate of non-Hispanic White (White) adults and was the highest rate observed among any racial and ethnic group during the pandemic. Compared with the Delta-predominant period, the proportion of unvaccinated hospitalized Black adults increased during the Omicron-predominant period. All adults should stay up to date (*1*) with COVID-19 vaccination to reduce their risk for COVID-19–associated hospitalization. Implementing strategies that result in the equitable receipt of COVID-19 vaccinations, through building vaccine confidence, raising awareness of the benefits of vaccination, and removing barriers to vaccination access among persons with disproportionately higher hospitalizations rates from COVID-19, including Black adults, is an urgent public health priority.

COVID-NET conducts population-based surveillance for laboratory-confirmed COVID-19–associated hospitalizations in 99 counties across 14 states.^§^ COVID-19–associated hospitalizations are those occurring among residents of a predefined surveillance catchment area who have a positive real-time reverse transcription–polymerase chain reaction (RT-PCR) or rapid antigen detection test result for SARS-CoV-2 during hospitalization or the 14 days preceding admission.

This analysis describes weekly hospitalization rates during Delta- and Omicron-predominant periods. Among nonpregnant and pregnant adults aged ≥18 years,^¶^ hospitalization rates were calculated overall, and by race/ethnicity and COVID-19 vaccination status. Age-adjusted rates were calculated by dividing the number of hospitalized COVID-19 patients by population estimates for race/ethnicity, and vaccination status in the catchment area. Vaccination status (unvaccinated, receipt of a primary series only, or receipt of a primary series plus a booster or additional dose) was determined for individual hospitalized patients and for the catchment population using state immunization information systems data (*2*).** Monthly incidence among adults who received booster or additional doses was calculated by summing the total number of COVID-19 patients with booster or additional doses hospitalized over all days of the month and dividing by the sum of adults with booster or additional doses in the underlying population for each day of the month.^††^ This method was also used for calculations in unvaccinated persons and those who received a primary series but not a booster or additional dose.^§§^

Using previously described methods (*3*), investigators collected clinical data on a representative sample of adult patients (7.9%) hospitalized during July 1, 2021–January 31, 2022, stratified by age and COVID-NET site. Surveillance officers abstracted data on sampled patients from medical charts. Pregnant women were excluded because their reasons for hospital admission (*4*) might differ from those for nonpregnant persons.

Variances were estimated using Taylor series linearization method. Chi-square tests were used to compare differences between the Delta- and Omicron-predominant periods; p-values <0.05 were considered statistically significant. Percentages presented were weighted to account for the probability of selection for sampled cases (*3*). Analyses were conducted using SAS statistical software survey procedures (version 9.4; SAS Institute). This activity was reviewed by CDC and conducted consistent with applicable federal law and CDC policy.^¶¶^

During the Omicron-predominant period, overall weekly adult hospitalization rates peaked at 38.4 per 100,000, exceeding the previous peak on January 9, 2021 (26.1) and the peak rate during the Delta-predominant period (15.5) (Figure 1). Age-adjusted hospitalization rates among Black adults peaked at 94.7 (January 8, 2022), higher than that among all other racial and ethnic groups, 3.8 times the rate among White adults (24.8) for the same week, and 2.5 times the previous peak (January 16, 2021) among Black adults (37.2). This was the highest age-adjusted weekly rate observed among any racial and ethnic group during the pandemic. During the Omicron-predominant period, hospitalization rates increased among unvaccinated persons and those who completed a primary series, with and without receipt of a booster or additional dose (Figure 2). Weekly rates among unvaccinated adults and adults who received a primary COVID-19 vaccination series with a booster or additional dose peaked at 149.8 (January 8, 2022) and 11.7 (January 22, 2022), respectively. The cumulative monthly age-adjusted hospitalization rate during January 2022 among unvaccinated adults (528.2) was 12 times the rates among those who had received a booster or additional dose (45.0) and four times the rates among adults who received a primary series, but no booster or additional dose (133.5). The rate among adults who received a primary series, but no booster or additional dose (133.5), was three times the rate among adults who received a booster or additional dose (45.0).

**FIGURE 1:**
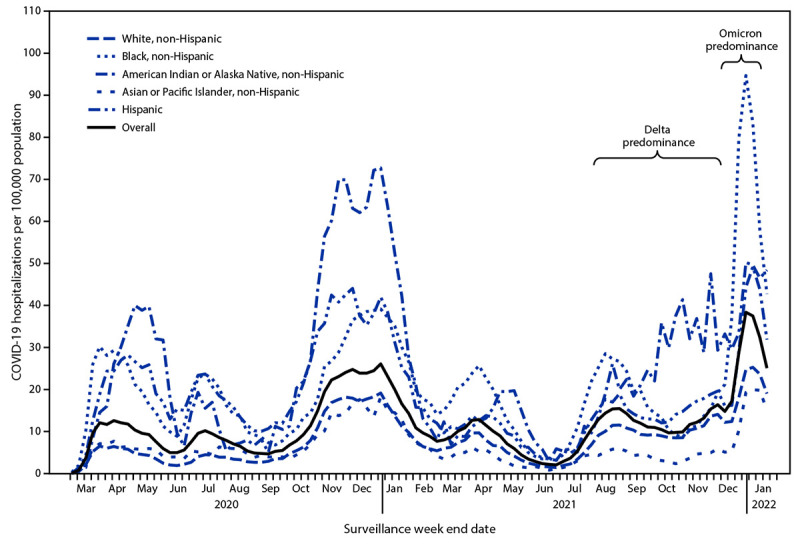
Weekly COVID-19–associated hospitalization rates* among adults aged ≥18 years, by race and ethnicity — COVID-19–Associated Hospitalization Surveillance Network, 14 states,^†^ March 2020–January 2022 * Overall rates are unadjusted; rates presented by racial and ethnic group are age-adjusted. **^†^** Selected counties in California, Colorado, Connecticut, Georgia, Iowa, Maryland, Michigan, Minnesota, New Mexico, New York, Ohio, Oregon, Tennessee, and Utah (https://www.cdc.gov/mmwr/volumes/69/wr/mm6915e3.htm). Starting the week ending December 4, 2021, Maryland data are not included in weekly rate calculations but are included in previous weeks.

**FIGURE 2:**
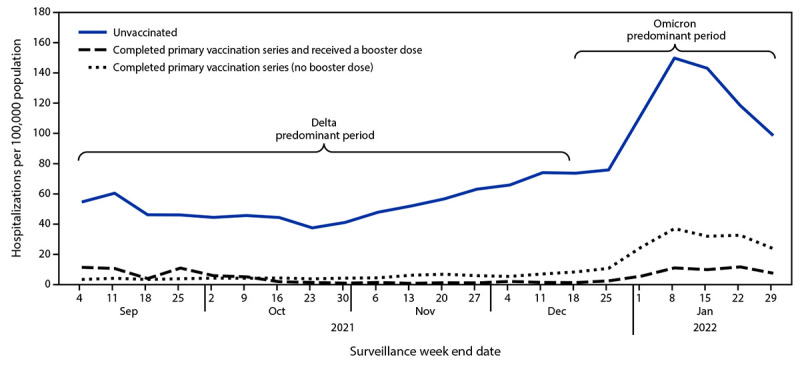
Weekly age-adjusted rates of COVID-19–associated hospitalizations among adults aged ≥18 years, by vaccination status* — COVID-19–Associated Hospitalization Surveillance Network, 13 states,^†^ September 4, 2021–January 29, 2022^§^ **Abbreviation:** COVID-NET = COVID-19–Associated Hospitalization Surveillance Network. * Adults who completed a primary vaccination series were defined as those who had received the second dose of a 2-dose primary vaccination series or a single dose of a 1-dose product ≥14 days before a positive SARS-CoV-2 test associated with their hospitalization but received no booster dose. Adults who received booster doses were classified as those who completed the primary series and received an additional or booster dose on or after August 13, 2021, at any time after completion of the primary series, and ≥14 days before a positive test result for SARS-CoV-2, because COVID-19–associated hospitalizations are a lagging indicator and time passed after receipt of a booster dose has been shown to be associated with reduced rates of COVID-19 infection (https://www.nejm.org/doi/full/10.1056/NEJMoa2114255). Adults with no documented receipt of any COVID-19 vaccine dose before the test date were considered unvaccinated. ^†^ Selected counties in California, Colorado, Connecticut, Georgia, Maryland, Michigan, Minnesota, New Mexico, New York, Ohio, Oregon, Tennessee, and Utah (https://www.cdc.gov/mmwr/volumes/69/wr/mm6915e3.htm). Iowa does not provide data on vaccination status. ^§^ Starting the week ending December 4, 2021, Maryland data are not included in weekly rate calculations but are included in previous weeks. To ensure stability and reliability of rates by vaccination status, data are presented beginning when 14 days have passed since at least 5% of the population of adults aged ≥18 years in the COVID-NET surveillance catchment area had received an additional or booster dose.

Clinical information was abstracted for 5,681 adults with COVID-19–associated hospitalization during July 1, 2021–January 31, 2022 (Table). Black adults accounted for a higher percentage of hospitalizations during the Omicron-predominant period (26.7%) than during the Delta-predominant period (22.2%, p = 0.05). Among all adults, relative to the Delta-predominant period, COVID-19–related illness was the primary reason for admission for a smaller percentage of hospitalizations (87.5% versus 95.5%, p<0.01), and median length of stay was shorter (4 versus 5 days, p<0.01) during the Omicron-predominant period; during this period, the proportion of patients admitted to an intensive care unit, who received invasive mechanical ventilation, and who died in-hospital decreased significantly (all p<0.01).

**TABLE:** Demographic characteristics and clinical interventions and outcomes in COVID-19–associated hospitalizations among nonpregnant adults aged ≥18 years (N = 5,681),* by vaccination status^†^ and period of SARS-CoV-2 variant predominance^§^ — COVID-NET, 14 states,^¶^ July 2021–January 2022

Characteristic	Variant predominance period, no. (%)								
	Total hospitalizations**			Vaccination status					
				Unvaccinated		Primary series, no booster		Primary series, plus booster	
	Delta (Jul 1–Dec 18)	Omicron (Dec 19–Jan 31)	p-value^††^	Delta (Jul 1–Dec 18)	Omicron (Dec 19–Jan 31)	Delta (Jul 1–Dec 18)	Omicron (Dec 19–Jan 31)	Delta (Jul 1–Dec 18)	Omicron (Dec 19–Jan 31)
**Overall^§§^**	**4,852 (64.1)**	**829 (35.9)**	**—**	**3,269 (71.8)**	**409 (28.2)**	**1,183 (58.0)**	**255 (42.0)**	**45 (15.3)**	**93 (84.7)**
**Median age, yrs, (IQR)**	60 (47–72)	64 (49–77)	<0.01	56 (43–67)	60 (46–77)	71 (61–80)	66 (52–78)	75 (69–82)	69 (59–79)
**Age group, yrs**									
18–49	1,419 (28.7)	251 (25.6)	0.01	1,185 (36.6)	141 (30.3)	140 (10.1)	71 (21.1)	2 (1.3)	13 (13.2)
50–64	1,723 (30.4)	265 (26.6)		1,274 (33.7)	142 (28.8)	310 (21.2)	77 (26.3)	7 (9.5)	23 (21.1)
≥65	1,710 (40.9)	313 (47.9)		810 (29.7)	126 (40.9)	733 (68.6)	107 (52.5)	36 (89.2)	57 (65.7)
**Sex**									
Men	2,574 (52.7)	435 (52.2)	0.83	1,751 (52.7)	225 (51.5)	610 (53.2)	127 (50.8)	21 (38.4)	50 (60.8)
Women	2,278 (47.3)	394 (47.8)		1,518 (47.3)	184 (48.5)	573 (46.8)	128 (49.2)	24 (61.6)	43 (39.2)
**Race/Ethnicity** ^¶¶^									
White, non-Hispanic	2,917 (54.4)	474 (47.6)	0.05	1,852 (50.2)	222 (40.7)	817 (63.1)	137 (46.4)	41 (87.9)	71 (70.8)
Black, non-Hispanic	943 (22.2)	185 (26.7)		687 (25.2)	98 (31.0)	169 (14.9)	60 (25.5)	3 (4.7)	11 (14.8)
American Indian or Alaska Native, non-Hispanic	63 (1.5)	8 (1.0)		46 (1.5)	5 (1.5)	15 (1.9)	3 (1.0)	0 (0.0)	0 (0.0)
Asian or Pacific Islander, non-Hispanic	133 (3.6)	19 (4.6)		88 (3.4)	9 (5.4)	36 (4.6)	7 (11.8)	0 (0.0)	3 (5.9)
Hispanic	589 (12.3)	43 (8.2)		447 (13.7)	52 (12.9)	101 (9.3)	33 (11.2)	1 (7.4)	6 (7.9)
LTCF residence***	264 (5.6)	53 (7.2)	0.18	76 (2.8)	14 (4.3)	155 (12.4)	24 (9.3)	9 (18.4)	11 (10.7)
**Any underlying medical condition** ^†††^	4,195 (88.5)	729 (91.0)	0.18	2,705 (85.1)	337 (87.7)	1,126 (96.8)	242 (96.3)	44 (99.1)	84 (89.6)
**Immunosuppressive condition** ^§§§^	505 (11.0)	132 (16.9)	<0.01	240 (7.7)	45 (10.4)	215 (18.6)	50 (21.7)	18 (44.7)	26 (69.5)
**Reason for admission**									
Likely COVID-19–related	4,487 (95.5)	712 (87.5)	<0.01	3,046 (96.3)	356 (89.5)	1,069 (93.0)	215 (85.3)	42 (94.4)	79 (85.5)
Inpatient surgery	33 (0.4)	12 (1.4)		14 (0.2)	4 (0.7)	17 (1.0)	5 (2.6)	0 (0.0)	2 (1.3)
Psychiatric admission requiring medical care	75 (1.5)	32 (3.9)		50 (1.6)	14 (3.5)	18 (1.3)	12 (4.7)	0 (0.0)	3 (5.1)
Trauma	69 (1.1)	23 (2.7)		37 (0.8)	13 (3.4)	27 (1.9)	5 (1.1)	1 (3.6)	2 (1.6)
Other	68 (1.3)	28 (4.1)		29 (0.8)	7 (2.6)	31 (2.6)	15 (6.3)	2 (2.0)	4 (5.2)
Unknown	13 (0.2)	3 (0.3)		7 (0.2)	2 (0.4)	6 (0.1)	0 (0.0)	0 (0.0)	1 (1.2)
**COVID-19–related signs or symptoms on admission** ^¶¶¶^									
Yes	4,503 (95.7)	739 (91.9)	<0.01	3,072 (97.0)	368 (93.6)	1,069 (92.9)	225 (90.3)	38 (89.5)	82 (90.6)
No	244 (4.3)	73 (8.1)		113 (3.0)	29 (6.4)	98 (7.1)	27 (9.7)	7 (10.5)	9 (9.4)
**Hospitalization outcome**									
Length of stay, days, median (IQR)	5 (3–10)	4 (2–9)	<0.01	5 (3–11)	5 (3–9)	5 (3–10)	4 (2–9)	6 (3–18)	4 (2–10)
ICU admission****^,††††^	1,148 (24.2)	149 (16.8)	<0.01	820 (25.3)	83 (17.4)	256 (22.7)	41 (16.1)	7 (21.1)	13 (16.8)
IMV^§§§§^	626 (13.6)	70 (7.6)	<0.01	467 (14.9)	36 (6.6)	124 (11.2)	21 (8.2)	5 (16.7)	6 (9.2)
In-hospital death^¶¶¶¶^	540 (12.6)	72 (7.0)	<0.01	385 (12.6)	42 (7.2)	123 (12.3)	19 (7.1)	5 (19.5)	7 (8.4)
Vaccination status*****									
Unvaccinated	3,269 (69.5)	409 (49.4)	<0.01	NA	NA	NA	NA	NA	NA
Primary series, no booster	1,183 (25.0)	255 (32.7)		NA	NA	NA	NA	NA	NA
Primary series, plus booster	45 (1.3)	93 (13.4)		NA	NA	NA	NA	NA	NA
**Days since last vaccination dose received before positive SARS-CoV-2 test result** ^†††††^									
15–60	NA	NA	NA	NA	NA	19 (0.9)	3 (1.1)	22 (52.9)	23 (31.2)
61–120	NA	NA		NA	NA	88 (7.7)	14 (7.6)	11 (30.8)	45 (49.3)
121–180	NA	NA		NA	NA	336 (26.6)	20 (5.9)	2 (6.3)	12 (13.9)
>180	NA	NA		NA	NA	560 (64.9)	183 (85.4)	8 (10.0)	4 (5.5)

**Abbreviations:** COVID-NET = COVID-19–Associated Hospitalization Surveillance Network; ICU = intensive care unit; IMV = invasive mechanical ventilation; LTCF = long-term care facility; NA = not applicable.

* Data are from a weighted sample of hospitalized nonpregnant adults with completed medical record abstractions and a discharge disposition. Sample sizes presented are unweighted with weighted percentages.

^†^ Vaccination status is based on state immunization information system data. Adults who completed a primary vaccination series were persons who had received the second dose of a 2-dose COVID-19 vaccination series or a single dose of a 1-dose product ≥14 days before a positive SARS-CoV-2 test associated with their hospitalization but received no booster or additional dose. Adults who received booster doses were classified as those who completed the primary series and received an additional or booster dose on or after August 13, 2021, at any time after completion of the primary series, and ≥14 days before a positive test result for SARS-CoV-2, as COVID-19–associated hospitalizations are a lagging indicator and time passed after receipt of a booster dose has been shown to be associated with reduced rates of COVID-19 infection (https://www.nejm.org/doi/full/10.1056/NEJMoa2114255). Adults with a positive result whose SARS-CoV-2 test date was ≥14 days after the first dose of a 2-dose series but <14 days after receipt of the second dose were considered partially vaccinated. Partially vaccinated adults, and those who received a single dose of a 1-dose product <14 days before the positive SARS-CoV-2 test result were not included in analyses by vaccination status but were included in rates and overall proportions that were not stratified by vaccination status. Adults with no documented receipt of any COVID-19 vaccine dose before the test date were considered unvaccinated. If the SARS-CoV-2 test date was not available, hospital admission date was used. Adults whose vaccination status had not yet been verified using the immunization information system data were considered to have unknown vaccination status and were included in total proportions but not stratified by vaccination status. Vaccination status is not available for Iowa and cases from Iowa are excluded from analyses that examined vaccination status. Additional COVID-NET methods for determining vaccination status have been described previously. https://www.medrxiv.org/content/10.1101/2021.08.27.21262356v1

^§^ Delta period: July 1, 2021–December 18, 2021, reflects the time when Delta was the predominant circulating variant; Omicron period: December 19, 2021–January 31, 2022, reflects the time when Omicron was the predominant circulating variant.

^¶^ Selected counties in California, Colorado, Connecticut, Georgia, Iowa, Maryland, Michigan, Minnesota, New Mexico, New York, Ohio, Oregon, Tennessee, and Utah (https://www.cdc.gov/mmwr/volumes/69/wr/mm6915e3.htm). Iowa does not provide data on vaccination status. Starting the week ending December 4, 2021, Maryland data are not included in calculations but are included in previous weeks.

** Total hospitalizations include data from selected counties in 14 COVID-NET states irrespective of vaccination status and includes adults with partial or unknown vaccination status. As a result, the number of total hospitalizations exceeds the sum of unvaccinated adults, adults who received a primary series without a booster or additional dose, and adults who received a primary series with a booster or additional dose.

^††^ Proportions between the pre-Delta and Delta period were compared using chi-square tests; p-values <0.05 were considered statistically significant, adjusted for multiple comparisons using the Bonferroni correction method.

^§§^ Percentages presented for the overall number are weighted row percentages. Percentages presented for demographic characteristics are weighted column percentages.

^¶¶^ If ethnicity was unknown, non-Hispanic ethnicity was assumed. Persons with multiple, unknown, or missing race accounted for 6.9% (weighted) of all cases. These persons are excluded from the proportions of race/ethnicity but are included in other analyses.

*** LTCF residents include hospitalized adults who were identified as residents of a nursing home/skilled nursing facility, rehabilitation facility, assisted living/residential care, long-term acute care hospital, group/retirement home, or other LTCF upon hospital admission. A free-text field for other types of residences was examined; patients with an LTCF-type residence were also categorized as LTCF residents.

^†††^ Defined as one or more of the following: chronic lung disease including asthma, chronic metabolic disease including diabetes mellitus, blood disorder/hemoglobinopathy, cardiovascular disease, neurologic disorder, immunocompromising condition, renal disease, gastrointestinal/liver disease, rheumatologic/autoimmune/inflammatory condition, obesity, feeding tube dependency, and wheelchair dependency.

^§§§^ Includes current treatment or recent diagnosis of an immunosuppressive condition or use of an immunosuppressive therapy during the preceding 12 months.

^¶¶¶^ COVID-19–associated signs and symptoms included respiratory symptoms (congestion or runny nose, cough, hemoptysis or bloody sputum, shortness of breath or respiratory distress, sore throat, upper respiratory infection, influenza-like illness, and wheezing) and non-respiratory symptoms (abdominal pain, altered mental status or confusion, anosmia or decreased smell, chest pain, conjunctivitis, diarrhea, dysgeusia or decreased taste, fatigue, fever or chills, headache, muscle aches or myalgias, nausea or vomiting, rash, and seizures). Symptoms are abstracted from the medical chart and might not be complete.

**** ICU admission and IMV are not mutually exclusive categories, and patients could have received both.

^††††^ ICU admission status was missing in 1.3% (weighted) of hospitalizations; these hospitalizations are included in other analyses.

^§§§§^ IMV status was missing in 1.4% (weighted) of hospitalizations; these hospitalizations are otherwise included elsewhere in the analysis.

^¶¶¶¶^ In-hospital death status was missing in 1.4% (weighted) of hospitalizations; these hospitalizations are otherwise included elsewhere in the analysis.

***** An additional 172 (3.4%, 95% CI = 2.7%–4.2%) adults were partially vaccinated, 69 (0.9%, 95% CI = 0.6–1.2) received a primary vaccination series <14 days before a positive for SARS-CoV-2 test result, and 186 (4.1%) had unknown vaccination status; these groups are not further described in this analysis.

^†††††^ If SARS-CoV-2 test date was missing, hospitalization admission date was used.

Among 829 adults hospitalized during the Omicron-predominant period, 49.4% were unvaccinated, compared with 69.5% during the Delta-predominant period (p<0.01). The proportion of hospitalized adults who received booster or additional doses increased from 1.3% during the Delta-predominant period to 13.4% during the Omicron-predominant period (p<0.01)***; among these, 10.7% were long-term care facility residents and 69.5% had an immunosuppressive condition.^†††^ Black adults accounted for 25.2% of all unvaccinated persons hospitalized during the Delta-predominant period; that proportion increased by 23%, to 31.0% during the Omicron-predominant period. Relative to the Delta-predominant period, the proportion of cases in non-Hispanic Asian or Pacific Islanders also increased, whereas the proportion in all other racial and ethnic groups decreased. The proportion of hospitalized Black adults who received a primary COVID-19 vaccination series with or without a booster or additional dose increased from 4.7% and 14.9%, respectively, during the Delta-predominant period to 14.8% and 25.5%, respectively, during the Omicron-predominant period; Hispanic adults experienced smaller increases.

## Discussion

During the period of Omicron predominance, hospitalization rates increased most sharply among Black adults in the United States relative to all other racial and ethnic groups examined and reached the highest rate observed among all racial and ethnic groups since the beginning of the pandemic. Relative to the Delta-predominant period, a larger proportion of hospitalized Black adults were unvaccinated. Although hospitalization rates increased for all adults, rates were highest among unvaccinated adults and lowest among adults who had received a primary series and a booster or additional dose. Hospitalization rates during peak Omicron circulation (January 2022) among unvaccinated adults remained 12 times the rates among vaccinated adults who received booster or additional doses and four times the rates among adults who received a primary series, but no booster or additional dose. The rate among adults who received a primary series, but no booster or additional dose, was three times the rate among adults who received a booster or additional dose. This is consistent with data showing the incidence of positive SARS-CoV-2 test results or death from COVID-19 is higher among unvaccinated adults and adults who have not received a booster than among those who have received a booster or additional dose (*5*).

Relative to the Delta-predominant period, a significantly shorter median length of hospital stay was observed during the Omicron-predominant period and smaller proportions of hospitalizations with intensive care unit admission, receipt of invasive mechanical ventilation, or in-hospital death. Other studies found similarly decreased proportions of severe outcomes among hospitalized patients with COVID-19 during this period (*6*).^§§§^

The prevalence of primary COVID-19 vaccination and of receipt of a booster dose were lower among Black adults compared with White adults. As of January 26, 2022, 39.6% of Black persons received a primary vaccine series; of those, 43.9% of adults received a booster dose once eligible. These proportions are lower compared with 47.3% of White persons who received a primary series and 54.5% of eligible adults who received a booster dose.^¶¶¶^ Relative to the Delta-predominant period, Black adults accounted for a larger proportion of unvaccinated adults during the Omicron-predominant period, and age-adjusted hospitalization rates for Black adults increased to the highest rate among all racial and ethnic groups for any week during the pandemic. A previous study conducted before the Omicron-predominant period that showed increased risk for COVID-19–associated hospitalization among certain racial and ethnic groups, including Black adults, and suggested the increased hospitalization rates were likely multifactorial and could include increased prevalence of underlying medical conditions, increased community-level exposure to and incidence of COVID-19, and poor access to health care in these groups (*7*). The increase in transmissibility of the Omicron variant might have amplified these risks for hospitalization, resulting in increased hospitalization rates among Black adults compared with White adults, irrespective of vaccination status. Taken together, these findings suggest that the increased risk for hospitalization among Black adults during the Omicron-predominant period might also be due, in part, to lower proportions of Black adults receiving both the primary vaccination series and booster doses.

The findings in this report are subject to at least four limitations. First, COVID-19–associated hospitalizations might have been missed because of hospital testing practices and test availability. Second, vaccination status is subject to misclassification; this might affect estimation of rates by vaccination status. Third, because immunocompromise status is not always known, it is not possible to distinguish between booster and additional doses; this could have influenced observed rates. Finally, the COVID-NET catchment areas include approximately 10% of the U.S. population; thus, these findings might not be nationally generalizable.

Coinciding with Omicron variant predominance, COVID-19–associated hospitalization rates among adults increased in late December 2021 and peaked in January 2022; rates increased more among Black adults relative to rates among adults of other racial and ethnic groups. Rates were highest among unvaccinated adults and lowest among those who had received a booster or additional dose. All adults should stay up to date (*1*) with COVID-19 vaccination to reduce their risk for COVID-19–associated hospitalization. Implementing strategies that result in the equitable receipt of COVID-19 vaccinations, though building vaccine confidence, raising awareness of the benefits of vaccination, and removing barriers to vaccination access among persons with disproportionately higher hospitalizations rates from COVID-19, including Black adults, is an urgent public health priority.

**Summary**

**What is already known about this topic?**

SARS-CoV-2 infections can result in COVID-19–associated hospitalizations, even among vaccinated persons.

**What is added by this report?**

In January 2022, unvaccinated adults and those vaccinated with a primary series, but no booster or additional dose, were 12 and three times as likely to be hospitalized, respectively, as were adults who received booster or additional doses. Hospitalization rates among non-Hispanic Black adults increased more than rates in other racial/ethnic groups.

**What are the implications for public health practice?**

All adults should stay up to date with COVID-19 vaccination to reduce their risk for COVID-19–associated hospitalization. Implementing strategies that result in the equitable receipt of COVID-19 vaccinations among persons with disproportionately higher hospitalizations rates, including non-Hispanic Black adults, is an urgent public health priority.

## Footnotes

*
https://covid.cdc.gov/covid-data-tracker/#variant-proportions

†
https://www.cdc.gov/coronavirus/2019-ncov/covid-data/covid-net/purpose-methods.html

§ Selected counties in California, Colorado, Connecticut, Georgia, Iowa, Maryland, Michigan, Minnesota, New Mexico, New York, Ohio, Oregon, Tennessee, and Utah (https://www.cdc.gov/mmwr/volumes/69/wr/mm6915e3.htm). Iowa did not provide immunization data but is included in the overall population-based hospitalization rates. Maryland did not contribute data after December 4, 2021, but did contribute data for previous weeks.

¶ Rates cannot be stratified by pregnancy status because the underlying population of pregnant women in the catchment area is unknown. Rates are calculated using the CDC National Center for Health Statistics’ vintage 2020 bridged-race postcensal population estimates for the counties included in surveillance. https://www.cdc.gov/nchs/nvss/bridged_race.htm

**
https://www.medrxiv.org/content/10.1101/2021.08.27.21262356v1

†† On August 13, 2021, CDC’s Advisory Committee on Immunization Practices (ACIP) issued the first of several recommendations for additional or booster doses of COVID-19 vaccine. Additional recommendations followed and data availability on booster-dose status varies by age because not all age groups were recommended by ACIP to receive booster doses at the same time. https://www.cdc.gov/mmwr/volumes/70/wr/mm7050e2.htm

§§ Adults who completed their primary COVID-19 vaccination series were defined as those who had received the second dose of a 2-dose primary vaccination series or a single dose of a 1-dose primary vaccine product ≥14 days before receipt of a positive SARS-CoV-2 test result associated with their hospitalization but received no additional or booster dose. Adults who received booster doses were classified as those who completed their primary vaccination series and received an additional or booster dose of vaccine on or after August 13, 2021, at any time after the completion of their primary series, and ≥14 days before a positive test result for SARS-CoV-2, because COVID-19–associated hospitalizations are a lagging indicator, and time passed after receipt of a booster dose has been shown to be associated with reduced rates of COVID-19 infection (https://www.nejm.org/doi/full/10.1056/NEJMoa2114255). Monthly incidence is based on SARS-CoV-2 positive test result date or, if not known, hospital admission date. Because the immune status of all patients is not known, an additional dose (recommended for persons with a weakened immune system) cannot be distinguished from a booster dose. This is a relevant consideration because vaccines can be less effective in persons with a weakened immune system. Persons who received only 1 vaccine dose of a 2-dose series ≥14 days before the SARS-CoV-2 test date or had received a single dose of either a 1- or 2-dose vaccination series <14 days before the positive SARS-CoV-2 test result were considered partially vaccinated and were not included in rates by vaccination status. Persons who received no doses of any COVID-19 vaccine were considered unvaccinated. The population of unvaccinated adults is determined by subtracting the number of adults who received any dose of vaccine, as previously defined, from the population. When possible, CDC associates a person’s primary vaccination series and booster dose with that person. However, linking is sometimes not possible because CDC does not receive personally identifiable information about vaccine doses. This can lead to overestimates of first doses and underestimates of subsequent doses, and underestimates of hospitalization rates in persons who received additional or booster doses. A continuity correction has been applied to the denominators by capping the percent population vaccination coverage at 95% by assuming that at least 5% of each age group would always be unvaccinated in each jurisdiction. This correction ensures that there is always a reasonable denominator for the unvaccinated population that would prevent hospitalization rates from growing unrealistically large because of potential overestimates of vaccination coverage. To ensure stability and reliability of rates by vaccination status, data are presented beginning 14 days after at least 5% of the age group-specific population of the COVID-NET surveillance catchment area has received an additional or booster dose. Additional COVID-NET methods for determining vaccination status have been described previously. https://medrxiv.org/cgi/content/short/2021.08.27.21262356v1

¶¶ 45 C.F.R. part 46, 21 C.F.R. part 56; 42 U.S.C. Sect. 241(d); 5 U.S.C. Sect. 552a; 44 U.S.C. Sect. 3501 et seq.

*** An additional 172 (3.4%, 95% CI = 2.7%–4.2%) adults were partially vaccinated, 69 (0.9%, 95% CI = 0.6–1.2) received a primary vaccination series <14 days before receiving a positive SARS-CoV-2 test result, and 186 (4.1%) had unknown vaccination status; these groups are not further described in this analysis.

††† Includes current treatment or recent diagnosis within the previous 12 months of an immunosuppressive condition or use of an immunosuppressive therapy.

§§§
https://www.medrxiv.org/content/10.1101/2022.01.11.22269045v1

¶¶¶
https://data.cdc.gov/Vaccinations/COVID-19-Vaccination-Demographics-in-the-United-St/km4m-vcsb
